# The relationship between social support and self-reported health status in immigrants: an adjusted analysis in the Madrid Cross Sectional Study

**DOI:** 10.1186/1471-2296-12-46

**Published:** 2011-06-08

**Authors:** Miguel Á Salinero-Fort, Laura del Otero-Sanz, Carmen Martín-Madrazo, Carmen de Burgos-Lunar, Rosa M Chico-Moraleja, Berta Rodés-Soldevila, Rodrigo Jiménez-García, Paloma Gómez-Campelo

**Affiliations:** 1 Fundación de Investigación Biomédica, Hospital Carlos III, (C/Sinesio Delgado, 10), Madrid (28029), Spain; 2 Servicio de Medicina Preventiva, Hospital Universitario Insular-Materno Infantil, (Avda. Marítima del Sur, s/n), Las Palmas de Gran Canaria (35016), Spain; 3 Unidad de Docencia e Investigación, Dirección Técnica de Docencia e Investigación, (C/Espronceda, 24), Madrid (28003), Spain; 4 Unidad de Epidemiología Clínica, Hospital Carlos III, (C/Sinesio Delgado, 10), Madrid (28029), Spain; 5 Unidad de Medicina Interna, Hospital Central de la Defensa Gómez Ulla, (Glorieta del Ejército, s/n.), Madrid (28047), Spain; 6 Departamento de Medicina Preventiva. Universidad Rey Juan Carlos, (Avda. de Atenas, s/n.), Alcorcón, Madrid (28922), Spain

**Keywords:** Social Support, Health Status Disparities, Immigrants, Spain

## Abstract

**Background:**

Social support is an important factor in the adaptation process of immigrants, helping for their integration in a new environment. The lack of social support may influence on well-being and health status. The aim of this study is to describe the social support of immigrant and native population and study the possible association between immigration and lack social support after adjusting for sociodemographic factors, income, stress and self-reported health status.

**Methods:**

Cross-sectional population based study of immigrants and national patients without mental disorders of 15 urban primary health centers in the north-eastern area of Madrid. Participants provided information on social support, stress level, perceived health status and socio-economic characteristics. Descriptive and multiple logistic regression were conducted.

**Results:**

The proportion of the global perception of social support among immigrants and natives was 79.2% and 94.2%, respectively. The lack of global social support adjusted prevalence ratio (PR) of immigrant was 2.72 (95% Confidence Interval = 1.81-4.09), showing a significant association with being male (PR = 2.26), having monthly income below 500 euros (PR = 3.81) and suffering stress (PR = 1.94). For the dimensions of lack of social support the higher association was being an immigrant and suffering stress.

**Conclusions:**

We conclude that with regardless of the level of monthly income, stress level, self-reported health status, and gender, immigrant status is directly associated with lack social support. The variable most strongly associated with lack social support has been monthly income below 500 euros.

## Background

Immigration is a recent phenomenon in Spain that has doubled during the second half of the nineties, marking a new social and political reality and raising many social and health challenges [1,2]. Currently, Spain's population has grown in more than 46 million people, nearly 12% (almost 17% in Madrid) is accounted by immigrants, and this percentage does not reflect the illegal fraction of these people who live within Spain's frontiers [3]. In the year 2010, in the north-eastern area of Madrid, most of the foreign population comes from Latin America, over 60% of foreigners are Ecuadorian, Colombian, Peruvian, Bolivian, Dominican and Paraguayan even reaching in some districts of this area above 80% [4]. These data are similar to those reported by other large cities in Spain [3].

Job insecurity, illegal status and legal instability, access difficulties to housing, social isolation and ethnic prejudice are just some of the many problems encountered by immigrants when they arrive in host country [5]. So, the migration experience includes major changes in the person's environment, with the incorporation of a new physical context, institutional and sociocultural (climatic and geographic changes in the rules and values), so it will be necessary to adjust to the new social position with a major transformation of its network of social relationships. All these circumstances may pose a risk for health, physical and psychological and make the process of adaptation and integration in the new society complex [6].

Given this multi-problem reality, social support ranks high among the factors that relate to the success of the migration and integration into the new society [7]. Therefore, in the migration experience, social support is a source of resources of different nature: providing affection (affective support), understanding and opportunities for social participation (positive social interaction support), providing information about the host country in the search of employment and housing (emotional/informational support), providing access to basic social resources, education and health, and assistance instrumental in areas such as language acquisition, processing or transport documents (instrumental support) [8].

Previous researches have confirmed the positive effect of social support and their functional dimensions, in the experience of migration on health and subjective well-being of immigrants, showing that isolated people reported less health, physical and psychological, instead and they found a positive relationship between social support and good perception of physical health and functional autonomy and improving subjective well-being [8-15]. In general, they seem to suggest that the mere presence of interpersonal bonds is insufficient, being essential to the sharing of resources of various types of social relations that may come to exert a positive impact on the health of immigrants. However the origins and mechanisms of the associations between immigration, social support and health status are unclear. These results highlight the need to clarify under what conditions social support is beneficial to the health and wellbeing of immigrants.

We hypothesize that the immigrant population would have worse social support than people born in Spain, regardless of monthly income and perceived health status.

The aim of this study is to describe the social support of immigrant and native population and study the possible association between immigration and lack of social support after adjusting for sociodemographic factors, income, stress and self-reported health status.

## Methods

### Patients

This is a multicenter cross-sectional descriptive study with a sampling frame that included all of the 20 primary care centers of Area 4 in Madrid, located in the north-eastern Area of Madrid (Spain). None of the sites had special programs targeted to immigrants. Finally, 15 urban primary care centers agreed to participate in the study. In this health area, data show that 18% of population is immigrant, of which 65% comes from Latin America [16].

Within each primary care center, we selected a sample of patients in which every nth patient (ranged from 75 to 125) was recruited to participate in the study, during the period from January 2007 to December 2009. Interviewers observed potential participants as they registered at the front desk for their physician's visit and selected every nth patient to approach for the study. The interviewer told the patient: "We are doing a study with population who were born in Spain or immigrants" and invited to participate in the study. To reduce screening and enrolling participants at multiple appointments, the interviewers ask whether the patient had "done this interview before".

Patients were eligible if they met the inclusion criteria and give and sign informed consent. Eligible patients were invited to learn more about the study in a private room. The interviewer explained that the aim of the study was "to describe the social support of immigrant and native population and examine the association between immigration and lack of social support and how they influence perceived health" and that responses were confidential.

Inclusion criteria to participate in the study were: older than eighteen years, who attended for a medical or nursing consultation, understood Spanish language. The study was approved by the Institutional Review Board of Ramón y Cajal Hospital (Madrid). Exclusion criteria in the study where: all those who refused to participate, patients with psychotic or mood disorder (bipolar type) and the patients with severe chronic diseases or significant physical or psychic disabilities.

### Methods

The interview was performed by two psychologists, who had received homogeneous training in interview methods and in the evaluation procedure of the study, in order to minimize interview bias between them. The course titled: 'The clinical interview' and 'The PI06/1407 Study', organized by 'Unidad de Formación e Investigación del Área 4 de Madrid', provided training in evaluation and in the identification of the sample and procedure of practical fieldwork of the study.

#### Variables in the study

The dependent variable was social support, assessed by the Medical Outcomes Study-Social Support Survey (MOS-SSS) which was developed by Shebourne and Stewart [17] and adapted and validated in the Spanish version by De la Revilla et al. [18] (Additional file 1). This is a brief, self-administered and multidimensional survey of 20 items, with a score ranging from 2 to 20 in a Likert scale ranking from 1 (never) to 5 (always), where a higher global score indicates the higher social support. The evaluated MOS perceived global social support and four dimensions of support: a) emotional/informational support as the expression of positive affect, empathetic understanding and the encouragement of expression of feelings/the offering of advice, information, guidance or feedback (items: 3, 4, 8, 9, 13, 16, 17 and 19); b) Positive social interaction support, the availability of others persons to have fun with you (items: 7, 11, 14 and 18); c) affective support, involving expressions of love (items: 6, 10 and 20); and d) instrumental support as availability to material aid or behavioral assistance (items: 2, 5, 12 and 15). To obtain an overall support index, were calculated the average of 19 items and for each subscale, calculate the average of the scores for each item in the subscale. Lack of social support was defined by less than 57 points and the cut off points suggested for lack of emotional, instrumental, social interaction and affective support were: 23/24, 11/12, 8/9 and 8/9, respectively [19]. In the study, Cronbach's Alpha for the total scale was 0.96 and for the subscales emotional/informational, positive social interaction, affective and instrumental support it was 0.94, 0.91, 088 and 0.89 respectively.

Stressful life was measured with the Social Readjustment Rating Scale (SRRS) by Holmes and Rahe [20] and translated and adapted for Spain by González de Rivera et al. [21] (Additional file 2). The scale includes a list of 43 items about high stress vital events as divorce, death in the family, job change, etc., during the last year. Items are scored ranking from 1 to 100. Stress is defined by values over 150 in the global score [22-24]. Cronbach's Alpha for SRRS scale was 0.81 in this study.

Self-reported health status was measured by a single-item self-report indicator: "Would you say your health in general is...?. Five response categories were combined into 3 categories: poor/fair, good or very good/excellent, as suggested by other authors [25] (Additional file 3). Native or immigrant status was based on the country where the person was born. Marital status was codified into four categories: single, married, divorced and widow. Occupational status was compressed in four categories: manager position, administrative/self employed, manual worker and unemployed. Monthly income was categorized as: less than 500 €, 500-1000 € and higher than 1000 € (Additional file 3). Additionally, in immigrant population we collected some specific data of the reasons and conditions for migration and years lived in Spain (Additional file 3).

The sample size was calculated for the worst support absence's expected prevalence: 50% (maximum possible uncertainty) in each subgroup (native and immigrant) and the following assumptions: 4% precision, 95% confidence interval, 20% beta risk and 20% loss. Calculated size was obtained by the IMIM (Municipal Institute for Medical Research) computer program GRANMO 5.2 and was 751 subjects in each group.

### Statistical analysis

Estimated descriptive statistics were mean and standard deviation (SD) for the quantitative variables, and frequencies for the qualitative variables. The corresponding frequency distributions of the qualitative variables were calculated, analyzing whether significant differences existed between both study populations (immigrants and native people). For the bivariate proportion comparisons, the Pearson chi-square method or the Fisher exact test method were applied. The Student's t-test was applied for the bivariate mean comparisons.

Multiple logistic regression was adjusted to examine the influence of migration status and social support (to ease our discussion, we consider this like a dichotomies dummy variable: yes/no), on self-reported health status, controlling for potential socio-demographic covariates that have shown relevant relationship in the specialized literature, such as age, marital status, gender, occupational status, monthly income and stress. Variables were introduced in the model step by step based on statistical significance in the bivariate analysis and relevance for the study. The interactions between migration status, sex and socioeconomic factors were also checked. Adjusted prevalence ratios (PR) with their corresponding 95% Confidence Interval (CI) were calculated.

In all instances, the accepted level of significance was 0.05 or less. Statistical analysis of the data was carried out with SPSS 15.0 (SPSS, Inc., Chicago, Illinois).

## Results

2258 patients (825 immigrants and 1433 natives) met the selection criteria for inclusion in the study and were invited to participate in this study. A total of 1515 subjects voluntarily participated in the study, 612 immigrants and 903 natives, giving an overall response rate of 67.1% (74.2% and 63%, respectively). The origin of this foreign population was 91% Latin American (16% Ecuatorian, 5.7% Peruvian and 5.5% Colombian), 6% European, 2% African and 1% Asian.

The socio-demographical characteristics of both populations are shown in Table 1. Statistically significant differences in the two subpopulations appear in age, educational level, occupational status, self-reported health status and monthly income. The majority of immigrants were in a legal situation of residence (85.2%), having Spanish nationality a quarter of them. The vast majority, 77.3%, were living with a family member and their main motive to immigrate was economic (63.5%), followed by family reassembly (31%). Mean length of residence in Spain was 6,7 (SD = 5.3 years).

**Table 1:** Socio-demographic variables of the study population

	Immigrant population (n = 612)	Spanish population (n = 903)	p-value
Age (SD^a^)	34.5 (9.5)	37.1 (11.3)	<0.01

Sex			
Women	74.0%	72.0%	0.38
Men	26.0%	28.0%	

Marital status			
Single	36.1%	41.5%	0.09
Married	53.8%	50.1%	
Divorced	8.7%	6.5%	
Widow	1.4%	1.9%	

Educational level			
No studies	1.1%	0.4%	< 0.01
Primary school	7.5%	10.6%	
High school	61.1%	43.6%	
Qualified	15.9%	25.8%	
Bachelor degree	14.4%	19.6%	

Occupational status			
Manager	1.5%	12.8%	< 0.01
Administrative/Self employer	12.1%	33.0%	
Manual worker	65.0%	27.2%	
Unemployed	21.4%	27.0%	

Monthly incomes			
> 1000 €	11.4%	3.0%	<0.01
500-1000 €	51.7%	19.5%	
< 500 €	36.9%	77.5%	

^a ^Standard deviation

Regarding occupational status before migration, 35% were administrative/self employed, 34% manual workers, 25% unemployed and 6% managers. The 5.5% of immigrant population reported having been victim of political violence and 8.5% of family violence.

**Figure 1:**
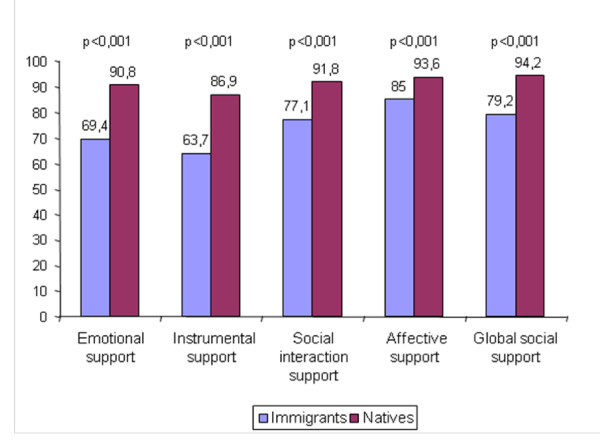
**Proportion of immigrants and natives with emotional, instrumental, social interaction, affective and global social support**.

There were significant differences in the perception of social support among immigrants and natives (p <0.001) for global social support and for the four dimensions studied (Figure 1). For global social support, 79% of immigrants compared to 94% of natives expressed receiving social support (PR = 4.28, CI = 3.05-6.03). The perception of emotional/informational, positive social interaction, affective and instrumental support is shown in Figure 1. As to social network size, the group of immigrants reported to have smaller networks than native patients (6 and 9 persons, respectively), showing a statistically significant difference (p <0.001).

With respect to the stress experienced in the past year, 55.4% of immigrants have had stress disorder while the natives have had it in a 45.6% (p <0.001). The analysis shows significant differences (p = 0.013) in self-reported health status. Natives have a better perception of excellent/very good health status than immigrants (34.7% versus 28.8%), there are practically no differences in good perception (47.6% versus 48.5%) and the poor/fair status is more frequent in immigrants (22.7% versus 17.7%).

Table 2 presents the global lack of social support adjusted prevalence ratios, showing a significant association with being an immigrant (PR = 2.72), male (PR = 2.26), having monthly income of 500-1000 € and <500 € (PR = 1.91 and PR = 3.81, respectively), suffering stress (PR = 1.94) and self-reported health status excellent/very good (PR = 0.46).

**Table 2:** Multivariable logistic regression of prevalence of lack of global social support adjusted by origin, age, sex, marital status, occupational status, income, stress and health status.

	**PR**^**a**^	**95% CI**^**b**^	p-value
Origin			
Native	1		
Immigrant	2.72	1.81-4.09	<0.01

Age (year)	1.03	1.01-1.04	<0.01

Sex			
Women	1		
Men	2.26	1.47-3.46	<0.01

Marital status			
Single	1		
Married	0.63	0.42-0.93	0.02
Divorced	1.57	0.90-2.74	0.11
Widow	1.42	0.49-4.10	0.52

Occupational status			
Manager	1		
Administrative/Self employer	3.39	0.77-14.83	0.10
Manual worker	5.34	1.25-22.76	0.02
Unemployed	3.32	0.76-14.53	0.11

Monthly income			
> 1000 €	1		
500-1000 €	1.91	1.27-2.88	0.02
< 500 €	3.81	2.12-6.87	<0.01

Stress			
No	1		
Yes	1.94	1.35-2.78	<0.01

Self-reported health status			
Poor/fair	1		
Good	1.17	0.77-1.78	0.46
Very good/Excellent	0.46	0.26-0.80	<0.01

^a ^Adjusted Prevalence Ratio. ^b ^95% Confidence Interval.

Considering the four dimensions of social support, it was noted that the lack of such support is significantly associated with being an immigrant and suffering stress, after adjusting for potential confounding variables, as shown in Table 3. However, the variable that explained a significant and greater association with the lack of social support prevalence was low income, especially income below 500 €/month (PR between 3 and 5).

**Table 3:** Multivariable logistic regression of prevalence of four dimensions of lack of global social support.

	Lack of emotional support			Lack of instrumental support			Lack of social interaction support			Lack of affective support		
	
	**PR**^**a**^	**95% CI**^**b**^	p-value	**PR**^**a**^	**95% CI**^**b**^	p-value	**PR**^**a**^	**95% CI**^**b**^	p-value	**PR**^**a**^	**95% CI**^**b**^	p-value
Origin												
Native	1			1			1			1		
Immigrant	2.81	2.01-3.91	<0.01	3.06	2.29-4.10	<0.01	1.92	1.34-2.76	<0.01	1.71	1.12-2.61	0.01

Age (year)	1.03	1.01-1.04	<0.01	1.02	1.01-1.03	0.02	1.03	1.02-1.05	<0.01	1.04	1.02-1.06	<0.01

Sex												
Women	1			1			1			1		
Men	0.72	0.52-0.99	0.05	1.35	0.98-1.85	0.06	1.02	0.71-1.47	0.92	0.55	0.37-0.83	<0.01

Marital status												
Single				1						1		
Married				0.76	0.56-1.02	0.07				0.39	0.25-0.60	<0.01
Divorced				2.37	1.47-3.82	<0.01				1.59	0.92-2.74	0.09
Widow				1.09	0.42-2.78	0.86				0.88	0.29-2.74	0.83

Occupational status												
Manager	1						1			1		
Administrative/self employer	1.13	0.52-2.46	0.76				1.38	0.55-3.45	0.49	4.78	1.11-20.66	0.03
Manual worker	1.81	0.86-3.80	0.13				2.3	0.95-5.56	0.06	6.36	1.49-27.13	0.01
Unemployed	1.04	0.48-2.25	0.94				1.08	0.43-2.71	0.87	2.54	0.57-11.40	0.22

Monthly income												
> 1000 €	1			1			1			1		
500-1000 €	1.93	1.30-2.34	<0.01	1.68	1.24-2.26	<0.01	2.15	1.49-3.09	<0.01	1.67	1.07-2.60	0.02
< 500 €	3.77	2.27-6.23	<0.01	3.33	2.05-5.41	<0.01	5	2.95-8.50	<0.01	3.57	1.90-6.70	<0.01

Stress												
No	1			1			1			1		
Yes	1.75	1.30-2.34	<0.01	1.42	1.08-1.85	0.01	1.92	1.39-2.65	<0.01	2.02	1.37-2.98	<0.01

^a ^Adjusted Prevalence Ratio. ^b ^95% Confidence Interval

## Discussion

This study has demonstrated that immigrant population attended in the north-eastern area of primary health care, compared with the natives, perceives a lower overall social support, which is also reflected in the four dimensions of social support (emotional, instrumental, social interaction, affective).

In a recent a study with the purpose to describe and compare immigrant (n = 46) and native Swedish patients (n = 46) in physical limitation, emotional state, social support and self-care [26], the authors observed that only the dimension of emotional social support was significantly worse (p = 0.048) in immigrants than Swedes, therefore immigrants had a greater need for emotional support that the natives. Another cross sectional study conducted in Spain compared quality of life of native Spanish's (n = 1009) and immigrants (n = 226) in the entire school population (12-18 year olds), excluded of the study those students who did not have enough knowledge of Spanish language to answer the questionnaire [27]. The results show that Spaniards had significantly high social support (42.2%) than immigrants (33.5%) (p = 0.02).

Moreover, these differences in social support are also shown in the size of the support network, where natives have averaged more than 9 members providing support and immigrants do not reach the average of 6 persons. These figures are lower than those found in other Spanish studies conducted with immigrant populations (9-10 members) [8] and even conducted with the Spanish population (6 members) [18].

Adittionaly, studies in the field of psychosocial sciences have described the social support characteristics of the immigrant population [8] but there are fewer studies that compare the social reality of the immigrant population with the natives in the area of health sciences [28]. To the best of our knowledge, in this respect our study would be an important contribution.

Regarding the possible variables associated with lack of perceived social support for immigrants, our data show that socioeconomic status, marital status, stress and self-reported health status are risk factors significantly associated with lack of social support. In a study conducted with people living in Canada, a strong correlation between the different dimensions of social support was identified; a positive correlation between physical health and perception of social support and a negative correlation between stress and low socioeconomic status were also found [29]. These results are consistent with the findings of our study, since the worst monthly income levels and stress were associated with lower global social support and its four dimensions.

In general, a better self-reported health status in the Spanish population than in immigrants has been found. These results are consistent with findings in other studies in our country, showing that the immigrant population perceives that their health is worse than the Spanish population [30]. As suggested in a previous research [31], one's perception of adequate social support is associated with better self-reported health status (excellent/very good health status reduce the prevalence of lack of social support by 54% [1-0.46]). So, the key factor to understanding social health in immigrants is social support.

Social networks have a direct effect on health, by the interaction with others and by social participation, which promotes healthier life behaviours and greater self-esteem and social competence [32]. This may explain the association between health perception status and social support of our population as observed in a previous study [33].

Moreover, our study has found that women have better perception of social support than men, except in the instrumental support. These results are coherent with other studies that have described higher perceived social support in women, especially in emotional and affective dimensions [34,35]. In several studies, the difference in perception of social support among both genders is due to various factors such as marital status, education level, age and socioeconomic conditions [36,37]. In this sense, our data demonstrate a relationship between perceived social support and marital status, so that being married is positively associated with social support and being divorced negatively, confirming the findings of other studies [11,38].

One of the sociodemographic characteristics with greater strength of association with the lack of social support is monthly income. A low level of income, below the minimum salary, is a stressful situation that favours the loss of social skills and increases stress. Both conditions reduce the availability of resources giving poor people less social support. This is also confirmed by Palomar and Cienfuegos results [39], in which after an analysis of variance and multiple regression, they found three socioeconomic levels (extreme poor, moderate poor and no poor) to explain the relationship between perception of social support and social and personal characteristics. They found that the extreme poor, compared with the other two groups, perceived little social support from family and friends, but like the moderate poor, perceived high levels of support from the people of the community in which they lived. For its part, the moderate poor, compared to extremely poor, reported a better support of children, and compared with non-poor, a better support of children and higher levels of perceived social support by neighbors.

Another study carried out by the Aragonese government (Spain) in 1993, which analyzed the interaction and social support in family units, eligible for economic assistance, only 47% of participants had social support, provided mainly by family and 6.9% lacked any type of social support. Women had more social interaction than men, as in our study, the relationship between socioeconomic status and social support was demonstrated [40].

The lack of social support is a variable with a known association with stress level, both are closely linked. Research has indicated that social support serves as a resource to minimize the negative effects of facing a stressful situation [39], such as migration to a foreign country. The results of this study are consistent with this line and suggest that people with a good perception of social support have lower level of stress in coping with stressful life situations.

Another variable of interest that can explain the degree of social support in the immigrant population is the time spent in the host country, a phenomenon already studied by Lin and Hung [41] that support the findings of our study, that shows the longer one lives in the country of migration the more social support it has. This relationship can be explained by several circumstances: a better understanding of the host language, a better sociocultural adaptation to the new society and by the immigrants' origin.

This study has some limitations. The main limitation is the exclusion of immigrants who did not have sufficient understanding of the Spanish language. To try to control it, it should have been conducted a transcultural adaptation to the different possible languages of immigrant assessment interview, although this was not done because the questionnaires used are validated for the Spanish population but not available in other languages. Therefore, not all immigrant groups in Spain are represented in the study population, as immigrants with poor language comprehension were not included in the study. Additionaly, it's possible that the desire (conscious or not) to please his physician with his participation in the study had included some patients with a poor understanding of the questions that have answers providing input and an unreliable, introducing an information bias in the study.

However, in general, the inmigrant population included for the study is representative of Spanish immigrants (according to statistics released by the Spanish Home Office and other public and private institutions), coming from latinoamerica, and less from Europe and North of Africa (Morocco). Another important limitation is the small size of some of the subpopulations (Asian, African and European). This forces a cautious interpretation and limits the ability to generalize the results to the examined groups of immigrant population.

Other limitation is the high number of women participants. This situation is due to the fact that women use primary health care services more often than men, as seen in the study of Esteban Peña and Health Survey of Castilla la Mancha (Spain), where the population of women studied was 67% and the use of primary health care services for women was 82%, respectively [42,43].

In addition, the cross-sectional design of this study limits the possibility of establishing causal relationships between variables.

Despite the limitations, this research offers an insight into personal and social factors that the immigrant population expressed as an important perceived lack of social support.

## Conclusions

We conclude that with regardless of the level of monthly income, stress level, self-perceived health and gender, immigrant status is directly associated with lack of social support. The variable most strongly associated with lack of social support has been monthly income below 500 euros. These results have to be taken into account for treatment and program planning for immigrants and when developing policies addressed at the immigrant population.

## Competing interests

The authors declare that they have no competing interests.

## Authors' contributions

MASF conceived the study and participated in its design and performed the statistical analysis and drafted the manuscript. LOS, CBL, RMCM drafted the manuscript and made substantial contributions to the analysis and interpretation. CMM participated in the design and coordinated the research group. RJG, BRS, PGC helped in the statistical analysis and drafted the manuscript. All authors read and approved the final manuscript.

## Pre-publication history

The pre-publication history for this paper can be accessed here:

http://www.biomedcentral.com/1471-2296/12/46/prepub

## Supplementary Material

Additional file 1 **Mos Social Support Survey**. The file includes the questionnaire to assess social support used in the study. Click here for file

Additional file 2 **Social Readjustment Rating Scale**. The file includes the questionnaire to assess stress used in the study. Click here for file

Additional file 3 **Sociodemographic and health status questionnaire**. The file includes the questionnaire to assess sociodemographic and health status variables used in the study. Click here for file
