# Supplementary material for: The relationship between social support and self-reported health status in immigrants: an adjusted analysis in the Madrid Cross Sectional Study
Source: BMC Fam Pract. 2011 Jun 8;12:46. doi: 10.1186/1471-2296-12-46 (PMC3129304; doi:10.1186/1471-2296-12-46)
Supplement: Additional file 1 — Mos Social Support Survey. The file includes the questionnaire to assess social support used in the study. [file 1471-2296-12-46-S1.DOC]

### Additional file 1 Title- Mos Social Support Survey.

| **MOS SOCIAL SUPPORT SURVEY** (MOS-SSS) Shebourne and Stewart, 1991 | | | | | |
| --- | --- | --- | --- | --- | --- |
| 1. About how many close friends and close relatives do you have (people you feel at ease with and can talk to about what is on your mind)? | | | | | |
| Write in number of close friends and close relatives: □ □ | | | | | |
|  | None of the time | A little of the time | Some of the time | Most of the time | All of the time |
| 2. Some to help you if you were confined to bed | 1 | 2 | 3 | 4 | 5 |
| 3. Someone you can count on to listen to you when you need to talk. | 1 | 2 | 3 | 4 | 5 |
| 4. Someone to give you good advice about a crisis | 1 | 2 | 3 | 4 | 5 |
| 5. Someone to take you to the doctor if you needed it | 1 | 2 | 3 | 4 | 5 |
| 6. Someone who shows you love and affection | 1 | 2 | 3 | 4 | 5 |
| 7. Someone to have a good time with | 1 | 2 | 3 | 4 | 5 |
| 8. Someone to give you information to help you understand a situation | 1 | 2 | 3 | 4 | 5 |
| 9. Someone to confide in or talk to about yourself or your problems | 1 | 2 | 3 | 4 | 5 |
| 10. Someone who hugs you | 1 | 2 | 3 | 4 | 5 |
| 11. Someone to get together with for relaxation | 1 | 2 | 3 | 4 | 5 |
| 12. Someone to prepare you meals if you were unable to do it yourself | 1 | 2 | 3 | 4 | 5 |
| 13. Someone whose advice you really want | 1 | 2 | 3 | 4 | 5 |
| 14. Someone to do things with to help you get you mind off things | 1 | 2 | 3 | 4 | 5 |
| 15. Someone to help with daily chores if you were sick | 1 | 2 | 3 | 4 | 5 |
| 16. Someone to share your most private worries and fears with | 1 | 2 | 3 | 4 | 5 |
| 17. Someone to turn to for suggestions about how to deal with a personal problem | 1 | 2 | 3 | 4 | 5 |
| 18. Someone to do something enjoyable with | 1 | 2 | 3 | 4 | 5 |
| 19. Someone who understands your problems | 1 | 2 | 3 | 4 | 5 |
| 20. Someone to love and make your feel wanted | 1 | 2 | 3 | 4 | 5 |
