# Supplementary material for: The relationship between social support and self-reported health status in immigrants: an adjusted analysis in the Madrid Cross Sectional Study
Source: BMC Fam Pract. 2011 Jun 8;12:46. doi: 10.1186/1471-2296-12-46 (PMC3129304; doi:10.1186/1471-2296-12-46)
Supplement: Additional file 2 — Social Readjustment Rating Scale. The file includes the questionnaire to assess stress used in the study. [file 1471-2296-12-46-S2.DOC]

### Additional file 2 Title – Social Readjustment Rating Scale.

| **SOCIAL READJUSMENT RATING SCALE** (RSSR) Holmes and Rahe, 1967 | |
| --- | --- |
| Indicate events that occurred in the past year | |
| 1. Death of spouse |  |
| 2. Divorce |  |
| 3. Marital separation |  |
| 4. Prison |  |
| 5. Death in the familiy |  |
| 6. Accident or illness |  |
| 7. Marriage |  |
| 8. Loss of job |  |
| 9. Reconciliation with spouse |  |
| 10. Retirement |  |
| 11. Illness in the family |  |
| 12. Pregnancy |  |
| 13. Sexual difficulties |  |
| 14. Birth in the family |  |
| 15. Change of work |  |
| 16. Change in frequency of arguments |  |
| 17. Death of close friend |  |
| 18. Change in line of work |  |
| 19. Change in frequency of arguments with spouse |  |
| 20. Purchase of house of high value |  |
| 21. Finished paying off loan |  |
| 22. Change of work responsibility |  |
| 23. Son/daughter leaves home |  |
| 24. Difficulties with police |  |
| 25. Recognition for outstanding professional word |  |
| 26. Spouse began or stopped working |  |
| 27. Began or stopped studying |  |
| 28. Addition or deduction in people living in the house |  |
| 29. Change of personal habits |  |
| 30. Difficulty with boss |  |
| 31. Change of work schedule |  |
| 32. Change of house |  |
| 34. change or creational activities |  |
| 35. Change of religious activities |  |
| 36. Change of social activities |  |
| 37. Purchase on credit of middle value |  |
| 38. Change in sleeping habits |  |
| 39. Change in the frequency of family gatherings |  |
| 40. Change in eating habits |  |
| 41. Vacation |  |
| 42. Christmas |  |
| 43. Fined for small infractions |  |
