# Supplementary material for: The relationship between social support and self-reported health status in immigrants: an adjusted analysis in the Madrid Cross Sectional Study
Source: BMC Fam Pract. 2011 Jun 8;12:46. doi: 10.1186/1471-2296-12-46 (PMC3129304; doi:10.1186/1471-2296-12-46)
Supplement: Additional file 3 — Sociodemographic and health status questionnaire. The file includes the questionnaire to assess sociodemographic and health status variables used in the study. [file 1471-2296-12-46-S3.DOC]

| **SOCIODEMOGRAPHIC QUESTIONNAIRE** | |
| --- | --- |
| **- Country of origin**  1.  Spain  2.  Latin America. Specify country………………  3.  Europe. Specify country………………  4.  Africa. Specify country……………… | - **Sex**  1.  Male  2.  Female |
| **- Marital status**  1.  Single  2.  Married  3.  Divorced  4.  Widow | - **Occupational status**  1.  Directive position  2.  Administrative/self employed  3.  Manual worked  4.  Unemployed |
| **- Monthly income**  1.  Less than 500 euros  2.  500-1000 euros  3.  Higher than 1000 euros | |
| **Only immigrants:** | |
| **- Length of residence in Spain**………………(years) | - **Migration status**  1.  Residence permit  2.  No Residence permit  3.  Nationalized  4.  Awaiting deportation, undocumented or visa expired |
| **- Who lives**  1.  Alone  2.  With family  3.  With friends  4.  Other Specify ……………… | - **Reasons for migration**  1.  Economic  2.  Political  3.  Studies  4.  Other Specify ……………… |
| **SELF-REPORTED HEALTH STATUS** | |
| **Would you say your health in general is….?**  1.  Poor  2.  Fair  3.  Good  4.  Very good  5.  Excellent | |

### Additional file 3 Title- Sociodemographic and health status questionnaire.
